# Architected Materials for Additive Manufacturing: A Comprehensive Review

**DOI:** 10.3390/ma15175919

**Published:** 2022-08-26

**Authors:** Nikolaos Kladovasilakis, Konstantinos Tsongas, Dimitris Karalekas, Dimitrios Tzetzis

**Affiliations:** 1 Digital Manufacturing and Materials Characterization Laboratory, School of Science and Technology, International Hellenic University, 57001 Thessaloniki, Greece; 2 Centre for Research and Technology Hellas, Information Technologies Institute (CERTH/ITI), 57001 Thessaloniki, Greece; 3 Laboratory of Advanced Manufacturing Technologies and Testing, University of Piraeus, Karaoli and Dimitriou 80, 18534 Piraeus, Greece

**Keywords:** additive manufacturing, architected materials, lattice structures, topology optimization, scaling laws

## Abstract

One of the main advantages of Additive Manufacturing (AM) is the ability to produce topologically optimized parts with high geometric complexity. In this context, a plethora of architected materials was investigated and utilized in order to optimize the 3D design of existing parts, reducing their mass, topology-controlling their mechanical response, and adding remarkable physical properties, such as high porosity and high surface area to volume ratio. Thus, the current re-view has been focused on providing the definition of architected materials and explaining their main physical properties. Furthermore, an up-to-date classification of cellular materials is presented containing all types of lattice structures. In addition, this research summarized the developed methods that enhance the mechanical performance of architected materials. Then, the effective mechanical behavior of the architected materials was investigated and compared through the existing literature. Moreover, commercial applications and potential uses of the architected materials are presented in various industries, such as the aeronautical, automotive, biomechanical, etc. The objectives of this comprehensive review are to provide a detailed map of the existing architected materials and their mechanical behavior, explore innovative techniques for improving them and highlight the comprehensive advantages of topology optimization in industrial applications utilizing additive manufacturing and novel architected materials.

## 1. Introduction

In the last decades, the industrial 3D design was evolved from two-dimensional sketches to 3D parametric models with the assistance of Computer-Aided Design (CAD). The next step in this evolution is the integration of the topology optimization procedure in the design and fabrication processes. The Topology Optimization (TO) process calculates the minimum amount of mass and its optimal distribution on an existing part for a specific set of applied loads, usually, that set is the operation conditions [1,2]. Thus, TO concentrates an increased scientific and industrial interest, especially in sectors such as the aeronautical, automotive, and biomechanical [3,4,5,6,7,8]. TO is not a new concept, it is existing since 1904 with Michell’s book about structural optimization [9], which expressed the first definition and investigated the mathematical formulation behind the TO. However, the practical implementation of TO occurs due to the development of sophisticated software, the increase of computational power, and the employment of advanced manufacturing systems, such as additive manufacturing methods and the integration of computer/robotics in machining processes. More specifically, additive manufacturing (AM), also known as 3D printing, is facilitating the TO procedures due to the rapid fabrication and the limited geometrical constraints of topologically optimized parts. Thus, TO has a strong dependency on AM capabilities [10].

In this context, TO has been deeply examined over the last decade and two different approaches have been developed: the density-based and the truss-based methods [11,12]. The density-based method, also known as generative design, has the ultimate objective to minimize the mass of a part, hence it is employed only for lightweight applications [13]. This approach divides the part’s volume into elementary elements (voxels) and performs Finite Element Analysis (FEA) for the applied loads. After the evaluation of the stresses for each voxel, an initial normalized relative density value is assigned for each voxel with values ranging from zero to one. A value of zero is assigned to voxels with zero or minimum stress concentration, which indicates that these voxels could be removed. A value of one is assigned to voxels with high and maximum stress concentration, which indicates the necessity of maintaining these voxels on the topologically optimized part. For all the other voxels, the normalized relative density has moderate values depending on stress concentration contours. Then, the algorithm removes the voxels with zero or with the lowest values of normalized relative density and the FE analysis is performed again. This procedure is repeated until the designer’s objective is achieved or one of the designer-assigned constraints is reached. The most widespread algorithms for density-based TO are the Solid Isotropic Material with Penalization (SIMP) algorithm, the Bi-directional Evolutionary Structural Optimization (BESO) algorithm, and level-set, which are employed by a plethora of commercial 3D design software [14,15,16].

On the other hand, the truss-based approach employs architected materials, also known as lattice structures, in order to topologically optimize an object [12]. This process has risen an increased scientific interest in the last decade and is the most complex method of TO. Again, the FE analysis possesses an essential role in this procedure in locating the stress concentration regions inside the part’s volume [17]. The solid material’s regions are replaced by lattice structures with specific relative density corresponding to the stress concentration for each region, i.e., for a region with low-stress concentration, a low relative density (5–10%) is applied, and for a region with high-stress concentration, a high relative density (>50%) is applied. However, this method has two major challenges in its implementation. The first is the selection of the applied architected material, as there is a vast number of different architected materials with unique geometry and unique mechanical and physical characteristics [18]. For a truss-based TO application, the properties of each architected material have to be evaluated in order to choose the proper lattice structure depending on the examined application. The second challenge is the size effect that affects all architected materials regardless of their geometry [19]. The size effect is the mechanism that shows how the mechanical behavior is compromised by the reduction of the relative density and drastically affects structures with relative densities below 50%. More specifically, the size effect influences the mechanical properties through a specific scaling law formula. According to the existing literature [19,20,21], the effective mechanical properties of every architected material are dependent on the applied relative density in exponential relation via the aforementioned scaling laws. Furthermore, lattice structures, with intense exponential relation (*n* > 2) between the mechanical properties and relative density, reveal low stiffness and strength with an extensive plateau after the yield point at the stress–strain diagram; this mechanical response is defined as bending-dominated behavior [22,23]. In contrast, architected materials with an almost linear relation (*n* ≈ 1) between the mechanical properties and relative density, show high stiffness and peak stress coupled with an intense post-softening effect after the yield point at the stress–strain diagram; this mechanical response is named as a stretching-dominated behavior [24,25]. All architected materials experience one of these two mechanical behaviors or a moderate response between these two.

Moreover, the truss-based TO process appears to be superior compared to the density-based approach, due to the fact that besides the lightweight design, it offers additional advantages to the structures, such as the high surface area to volume ratio, high porosity, etc. Hence, the current review aims to present the up-to-date research on architected materials and their potential applications. More specifically, the definition and the physical properties of the architected materials are presented in-depth in Section 2 coupled with a profound classification of the lattice structures based on their geometry and their mechanical behavior. Section 3 focuses on the most widespread methods of optimizing the mechanical performance of the existing architected materials that have emerged through the literature in the last decade. In Section 4, an analysis of their main mechanical properties is presented and a comprehensive review of the existing literature on the architected materials’ mechanical response is conducted. The selection criteria of the presented research focused on the impact of the relative density of the architected materials on the effective mechanical properties via scaling laws through extensive quasi-static experiments. This process creates a mechanical behavior library for the architected materials in order to facilitate their implementation in industrial applications. Finally, in Section 5, the main applications of topology optimized parts utilizing architected materials and additive manufacturing techniques are listed. The ultimate objective of this comprehensive review is to provide a detailed guide about architected materials and how to implement them in commercial applications utilizing additive manufacturing technologies.

## 2. Theoretical Background and Classification of Architected Materials

### 2.1. Theoretical Background of Architected Materials

Architected materials, also known as architectured materials, are a class of materials that modify the physical and mechanical properties of the overall structure due to their unique topology and geometry. The first architected materials were detected in nature in form of foams and cellular materials, such as bones, corals, etc. [26]. It was observed that they possessed special characteristics such as lightweight structures and topologically controlled mechanical properties. Thus, the imitation of natural cellular materials was employed by copying the observed natural structures or developing new artificial architected materials, known as lattice structures.

The most crucial physical property of lattices is the relative density. The relative density of an architected material is the ratio of the lattice volume (*V_lattice_*) to the volume of the bounding box (*V_bb_*) that includes the whole structure, and it is emerged by the Equation (1). Where *ρ_solid_* and *m_solid_* express the density and the mass of a solid item with a volume equal to the bounding box volume. In addition, *ρ_material_* and *m_lattice_* represent the material’s density and the mass of the lattice structure, correspondingly.

(1) $$\bar{\rho }=\frac{\rho_{lattice}}{\rho_{solid}}=\frac{\frac{m_{lattice}}{V_{bb}}}{\frac{m_{solid}}{V_{bb}}}=\frac{m_{lattice}}{m_{solid}}=\frac{\rho_{material}\cdot V_{lattice}}{\rho_{material}\cdot V_{bb}}\to \bar{\rho \left(l,t\right)}=\frac{V{\left(l,t\right)}_{lattice}}{V{\left(l\right)}_{bb}}$$

Depending on the applied relative density, the architected materials are characterized as foam or lattice structures and their mechanical properties are modified. More specifically, when the structures have an ultra-low relative density (<5%) are defined as foams and reveal hyper-elastic non-linear behavior with limited structural integrity regardless of the construction material. Thus, these architected materials could be utilized for impact and high-strain applications. On the other hand, when the relative density is between 10–50%, the architected materials are defined as lattice structures, and depending on the lattice geometry and the exact percentage of relative density, they could be used for structural or energy absorption applications. It is worth mentioning that the architected materials with relative densities above 60% have mechanical behavior close to solid objects [19].

The relative density of the periodic architected materials is strongly connected with the ratio between the thickness (*t*) of the strut (for strut-lattices) or the wall (for sheet/surfaces) to the length (*l*) of the structure’s unit cell. On the other hand, in stochastic structures, the relative density is dependent on the ratio of the strut/wall thickness to the distance between the random seeds of the stochastic structure. According to the existing literature [27,28,29], these ratios influence the relative densities of the architected materials in different ways depending on the geometry of the structures. More specifically, for the majority of sheet-TPMS lattices, the *t*/*l* ratio appeared to have a linear relation with the relative density, as is shown in Equation (2). In contrast, the majority of the 2.5D lattices (honeycombs, etc.) and the 3D strut lattices (octet, diamond) influence the relative density with a second or third order polynomial mathematical formulation depending on the structure’s geometry and the accuracy of the calculations, as presented in Equations (3) and (4). It is worth mentioning that the *C*_1_, *C*_2_, and *C*_3_ are constants that are calculated according to the applied architected material. Moreover, *t* is always lower than the *l*, often *t* « *l*, which leads to *t*/*l* < 1. Thus, in strut lattices, the relative density is increasing exponentially when the *t*/*l* is low in contrast with the sheet-TPMS lattices, in which the relative density is increasing linearly.

(2) $$\bar{\rho }=C_{1}\cdot \frac{t}{l}$$

(3) $$\bar{\rho }=C_{2}\cdot {\left(\frac{t}{l}\right)}^{2}$$

(4) $$\bar{\rho }=C_{2}\cdot {\left(\frac{t}{l}\right)}^{2}-C_{3}\cdot {\left(\frac{t}{l}\right)}^{3}$$

### 2.2. Classification of Architected Materials Based on the Geometry

**Figure 1:**
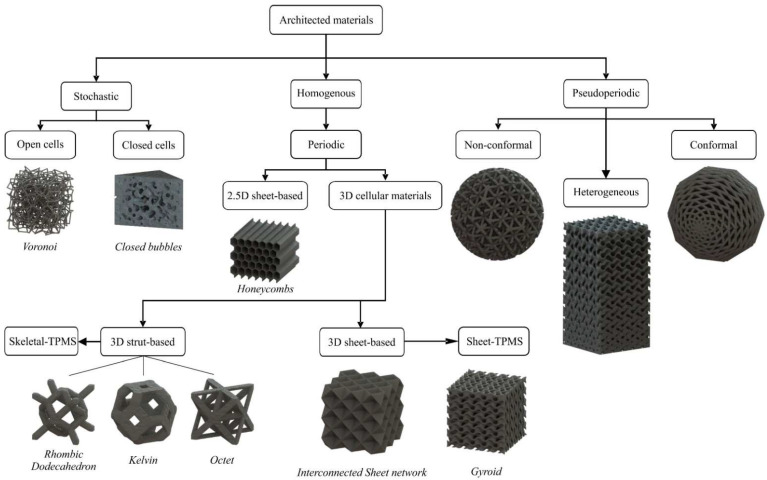
Classification of architected materials based on the geometry.

There is a vast number of architected materials with different geometries, thus it is necessary to classify them into categories based on their basic topological characteristics, as is shown in Figure 1. The first criterion is the periodicity of the structure, hence three families of structures are derived from this: the stochastic, the periodic, and the pseudo-periodic [30].

Stochastic architected materials are cellular materials without repeating elementary geometry, i.e., the unit cell and their geometry is derived from random functions and equations [31,32,33]. All the natural architected materials are stochastic, as they are not characterized by a unit cell. There are also artificial stochastic architected materials, which are designed with the assistance and combination of random functions and algorithms of topology, such as Voronoi, Delaunay, etc., in order to mimic the natural cellular materials. Furthermore, the stochastic architected materials are divided into open and closed cells, as is presented in Figure 1. Stochastic has commonly consisted of closed cells, i.e., multiple close volumes of air within the overall structure’s volume. It is worth mentioning that 3D printing enabled the manufacturing and examination of artificially stochastic lattice structures with high precision and low relative densities [34].

Periodic architected materials are the majority of fabricated and examined artificial cellular materials. The first reason is the ease of design due to the periodicity, i.e., when the geometry of one unit is designed then it could be repeated in the three dimensions. The second reason is the predictability of their mechanical properties, as the symmetric of the structure could be utilized via periodic boundary conditions (PBC) in order to simulate their mechanical response [35]. As it is illustrated in Figure 1, the periodic cellular material could be 2.5D or 3D [30]. The 2.5D structures are the simplest form of periodic architected materials and consist of sheet networks, which are designed from 2D geometrical shapes that are extruded in the third dimension, such as the honeycombs. Moreover, the 3D periodic architected materials are either strut or sheet interconnected networks/surfaces. There is a plethora of strut lattices; some of the most widespread are the Octet, Kelvin, and Rhombic Dodecahedron, which are portrayed in Figure 1. The sheet cellular materials are separated into shell lattices and triply periodic minimal surfaces (TPMS). The shell lattices are attained when plates or surfaces are positioned in certain positions, which are usually derived from modifying strut 3D structures, as is shown in Figure 1. On the other hand, TPMS structures follow specific trigonometric equations that control their geometry and have the unique topology characteristic of a mean curvature equal to zero at every point of the surface [36]. For this reason, TPMS structures have a higher surface area to volume than the rest and are also self-supported structures allowing their fabrication through the additive manufacturing processes [37].

Finally, the pseudo-periodic architected materials consist of either 2.5D or 3D cellular materials that have a variable relative density or an interaction with the boundaries of the structure [38]. In the first case, the relative density’s variation occurs by changing the periodicity of the structures, i.e., changes in the length of unit cells or from changes in the strut/wall structures. These structures are also called heterogeneous lattice structures. It is worth noting that when the relative density of a structure increases gradually, in order to distribute the receiving loads uniformly, then the structure is named a functionally graded lattice structure [39]. The second case occurs when the lattice structure follows the overall structure’s boundary, also known as conformal, or when the lattices are interrupted by the overall structure’s boundary, also known as non-conformal.

### 2.3. Classification of Architected Materials Based on the Mechanical Response

Existing literature has proven that the effective mechanical properties of an architected material are dependent on the relative density of the structure complying with the previously mentioned scaling law [19,20,40]. After extensive research, two major mechanical behaviors have been observed based on the exponent of the scaling law for the effective elastic modulus of a lattice structure and the curve of engineering stress to strain diagrams: the stretching-dominated and the bending-dominated behavior.

**Figure 2:**
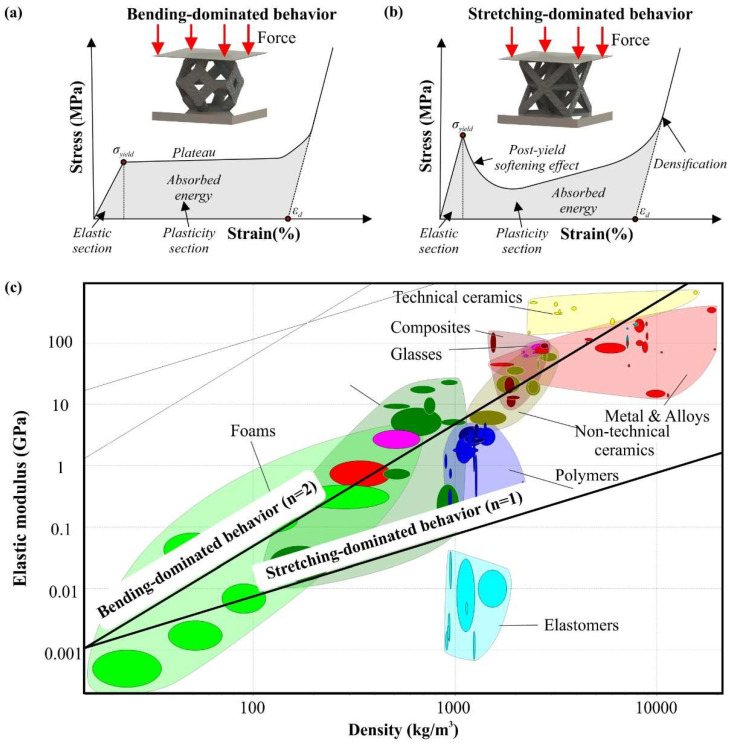
Indicative mechanical response for a structure with: (**a**) stretching-dominated behavior (Octet); (**b**) bending-dominated behavior (Kelvin); (**c**) Material property chart coupled with the curve for the two examined mechanical behaviors.

Stretching-dominated behavior occurs in architected materials with high connectivity, either with struts or with surfaces, and limited degrees of freedom in each joint/node leading to many possible self-stress situations [24]. In loading conditions, the first response of the structure is to elastically stretch the struts/surfaces; thus, the effective elastic modulus has a linear relation with the applied relative density, as is shown in Equation (5), and the exponent of the scaling law is equal with one (*n* = 1). Additionally, Figure 2a portrays an indicative stress–strain diagram for a stretching-dominated behavior.

(5) $$\frac{E_{l}}{E_{s}}=G_{1}\cdot \bar{\rho }$$

where *E_l_* is the effective elastic modulus of the applied architected material, *E_s_* is the elastic modulus of solid material, and *G*_1_ is a constant that is dependent on the geometry and the material of the applied lattices. The architected materials with stretching-dominated behavior or close to stretching-dominated behavior (*n* ≈ 1) are stiffer than the lattices with bending-dominated behavior and reveal higher peak strength due to their connectivity. However, the intense loading of the struts/surfaces at the peak strength leads to plastic buckling or brittle fracture of the majority of them, rapidly decreasing the strength of the structure. This phenomenon is known as the post-softening effect and it appears in the stress to strain diagram with a deep drop of the stress–strain curve after the peak stress, as portrayed in Figure 2a. Hence, the lattices with stretching-dominated behavior are excellent candidates for applications that require enhanced structural integrity. Nevertheless, these structures appear to have moderate performance in terms of energy absorption due to the post-softening effect, which results in most of the energy being absorbed within the elastic section.

The bending-dominated behavior appears in lattice structures with low connectivity and allows the struts/surfaces inside the structures to bend upon the application of loads. Due to the bending of the struts/surfaces, elastic deformation is governed by the surface’s second moment of inertia, which leads to exponential relation between the effective elastic modulus and the relative density. The exact mathematical formula is presented in Equation (6), where *G*_2_ is a constant that is dependent on the geometry and the material of the lattices, and n is an exponent with a value equal to or higher than two (*n* ≥ 2). In addition, Figure 2b depicts an indicative stress–strain diagram for a bending-dominated behavior.

(6) $$\frac{E_{l}}{E_{s}}=G_{2}\cdot {\left(\bar{\rho }\right)}^{n}$$

The architected materials with bending-dominated behavior reveal low stiffness and peak strength due to the bending of struts/surfaces and the low connectivity of the structure’s elements. However, after the peak stress and due to the bending loading condition of the struts/surfaces of the lattice structure, these structures continue to receive constant stresses creating a plateau section in the stress to strain diagrams as is shown in Figure 2b. During the plateau, the structure is deformed plastically until the densification section (after 50% of strain). This plastic deformation leads to remarkable amounts of energy absorption from the plastic section. Thus, the architecture materials with bending-dominated mechanical behavior have high crashworthiness and are suitable for impact applications, such as helmets, armors, etc. [41,42]

As it was presented, the connectivity of an architected material’s unit cell has an essential role in the mechanical behavior of the structure. Hence, for the periodic strut and 2.5D lattices, Maxwell established a mathematical regulation, known as Maxwell’s stability criterion, which defines if one architected material has stretching-dominated or bending-dominated behavior by the number of the consisted struts/beams (*b*) and nodes/joints (*j*), according to the Equations (7) and (8) [24,43]. It is worth noting that this classification based on mechanical behavior is valid only for architected materials with fixed nodes/joints, in other cases the structure is a mechanism. In Figure 2c, an indicative material diagram is presented coupled with the curves of elastic modulus to density for both examined mechanical responses.

(7) $$For\;2D\;structures:M=b-2j+3\left\{\begin{array}{c} if\;M<0\;bending-dominated\;behavior\\ \;if\;M\geq 0\;stretching-dominated\;behavior \end{array}\right.$$

(8) $$For\;3D\;structures:M=b-3j+6\left\{\begin{array}{c} if\;M<0\;bending-dominated\;behavior\\ \;if\;M\geq 0\;stretching-dominated\;behavior \end{array}\right.$$

**Figure 3:**
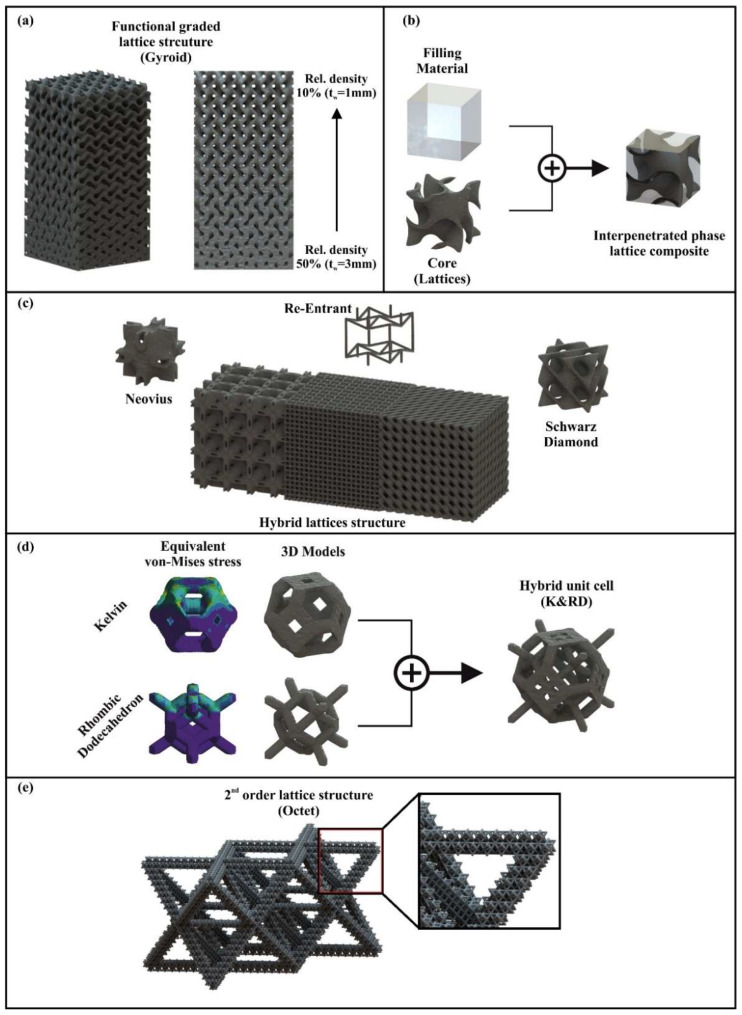
Optimization processes of architected materials: (**a**) Functional gradation; (**b**) Interpenetrating phase composites with architected material; (**c**) Hybridization of a structure; (**d**) Hybridization of a unit cell; (**e**) Lattice structure with higher-order.

Finally, one family of the most interesting architected materials is the auxetic lattice structures, such as the Re-Entrant structure (Figure 3). The auxetic structures reveal similar stress to the strain diagram with the bending-dominated structures, but they have the unique mechanical characteristic of a negative Poisson ratio. A negative Poisson ratio is expressed in compression loading with intense deformation/reduction of the dimensions, which are perpendicular to the direction of loading. In cases of tensile loading, the auxetic structures exhibit large deformations with relatively low stress (below the yielding stress). Due to these mechanical characteristics, the auxetic materials are an essential tool for 4D printing applications in which the applied stresses (mechanical, thermal, etc.) lead to a significant change in the structure’s dimensions, i.e., modification of the entire structure’s shape [44,45,46].

## 3. Optimization of Architected Materials

The architected materials are employed in order to structurally optimize a part’s geometry by reducing the mass and providing a more uniform mass distribution. However, the implementation of architected materials could be enhanced by a series of methods that exploited the unique topological characteristics of the cellular materials. According to the existing literature [39,40,47,48,49,50], there are five widespread methods to optimize architected materials and lattice structures, which are also areas of extensive scientific interest.

The first and the most widespread method is named functionally gradation of the lattice structure. This technique is second-level topology optimization of an object, as in the first level the replacement of solid region by lattice structure was performed. In this second-level topology optimization, the relative density of a lattice structure is modified depending on the loads that the structure receives. In detail, for a specific application, the regions with low-stress concentration employ lattice structures with lower relative density in contrast with the regions with higher stress concentration [51]. In this way, the mechanical performance of the lattice structure is improved without changing the overall mass, as the mean relative density of the structure remains the same. The increment of the relative density in the structure could be employed rapidly, for example increasing from 10% to 60% relative density for two contiguities regions; or it could be performed gradually with linear, exponential, or scalar-field mode providing a smoother transaction between the regions of low and high relative densities. It is worth mentioning that the changes in relative density are achieved by changing the *t*/*l* ratio of the lattice structure, either by shifting the numerator or the denominator or even both; however, the most common way is by changing the lattices’ strut/wall thickness (numerator-*t*). Figure 3a illustrates an indicative implementation of functional graded architected materials (Gyroid) for impact and energy absorption experiments.

The second optimization method for the architected materials requires the utilization of one extra manufacturing process besides the additive manufacturing. The concept of this procedure is to replace the air inside the volume of an architected material with another material, as it is presented in Figure 3b. With this process, solid composite structures are created consisting of two materials the architected material and the filling material; these structures are also known as interpenetrating phase composites. There is a wide variety of filling materials from epoxy and silicone rubber compounds to polymer foams, such as polyurethane. Furthermore, the interpenetrating phase composites with architected materials could produce composite materials with unique physical and mechanical properties. For example, lattice structures with high electrical conductivity [52] or lattice structures with higher energy absorption rates [53].

The third technique is also very common among the research community and deploys two or more different architected materials inside the overall structure of a part. This method is also known as the hybridization of lattice structures [47]. More specifically, a different selection of architected materials is performed for a different region of the part with the objective to improve the mechanical response of the structure, but also to exploit the unique physical, mechanical, or aesthetic characteristics of the applied architected materials [54]. For example, auxetic architected materials could be deployed in regions where tensile stresses appear, and sheet-TPMS, such as the Schwarz Diamond structure, could be employed in the rest of the body in order to secure the structural integrity of the part. In addition, the hybridization lattice structures could be utilized in impact applications with remarkable results. This is achieved by maximizing the energy absorption of the whole structure with the combination of a bending-dominated architected material, which receives the maximum plastic deformation, with a stretching-dominated lattice structure, which receives elastic deformation with high peak strength. As in the functional gradation case, the hybridization process of a structure could be performed rapidly or gradually. Figure 3c illustrates a hybrid lattice designed for bending and energy absorption tests.

The fourth method fundamentally changes the unit cell structure of an architected due to the fact that it combines two or more architected materials in the volume of one unit cell in order to produce enhanced cellular material in terms of mechanical behavior [55]. Again, this is a hybridization method like the previous one with the difference that is performed at a level of a unit cell. In this method, the hybridization of the distinct cellular materials is performed by assessing the individual mechanical performance of two different architected materials and combining them. More specifically, the stress concentration regions of each structure are located, and the architected materials are combined in such a way to enhance and reinforce these regions leading to a new architected material with improved mechanical behavior. This method creates advanced cellular material with high geometry complexity that could be combined with the two aforementioned optimization methods of lattice structures for applications with high demands of structural integrity. Figure 3d shows unit cells of the hybrid architected materials coupled with the initial cellular materials and their contours of von-Mises stress which led to the proper combination of these structures.

The last technique leads to even more complex lattice structures, as it produces architected materials with higher-order structures [56]. In detail, a lattice structure is embedded within the elements of an architected material, i.e., strut/walls and nodes. Thus, these elements consist of lattice structures, as is portrayed in Figure 3e. This method is utilized in order to produce ultra-lightweight structures and structures with ultra-high porosity. However, the major challenge of this optimization method is the manufacturing procedure, as the lattice structures within the strut/surface volume are one order smaller than the whole structure. Thus, a 3D printer with sufficient build volume and high accuracy, such as the Selective Laser Melting (SLM) technique, is required, or a 3D printer with an ultra-high accuracy, such as the two-photon polymerization additive manufacturing technique.

## 4. Mechanical Properties of Architected Materials

The architected materials are employed in order to structurally optimize a part’s geometry by reducing the mass and providing a more uniform mass distribution. In the previous sections, the theoretical background of architected materials coupled with the existing types of their classification were discussed, based on their geometry and on their mechanical behavior. Moreover, the most widespread methods of optimizing architected materials were analyzed. In this section, the mechanical behavior of architected materials is discussed, and numerical data of the mechanical properties are presented based on experiments and measurements from the existing literature.

One of the major disadvantages of architected materials is the anisotropy of their mechanical properties, due to the existence of extensive porosity. In other words, the majority of architected materials reveal different mechanical properties in different directions; this could be catastrophic in commercial applications. Thus, the measurement of anisotropy is the first analysis, when a new architected material is created. According to the literature [57,58], the anisotropy measurement of architected materials is performed on one unit cell of the chosen structure and could be evaluated with two methods. The first method is only applied to architected materials with cubic external volume and utilizes the Zener anisotropy ratio. The Zener ratio (*A_r_*) is a positive dimensionless number that quantifies the anisotropy of an architected material with values above zero and up to one for completely isotropic material. The Zener ratio is calculated from the constants (*C*) of the following stiffness matrix and Equation (10).

(9) $$\underline{C}=\left[\begin{array}{cc} \begin{array}{ccc} C_{11} & C_{12} & C_{12}\\ C_{12} & C_{11} & C_{12}\\ C_{12} & C_{12} & C_{11} \end{array} & \begin{array}{ccc} 0 & \;0 & \;0\\ 0 & \;0 & \;0\\ 0 & \;0 & \;0 \end{array}\\ \begin{array}{ccc} 0 & \;0 & \;0\\ 0 & \;0 & \;0\\ 0 & \;0 & \;0 \end{array} & \begin{array}{ccc} C_{44} & 0 & 0\\ 0 & C_{44} & 0\\ 0 & 0 & C_{44} \end{array} \end{array}\right]$$

(10) $$A_{r}=\frac{2\cdot C_{44}}{C_{11}-C_{12}}$$

**Figure 4:**
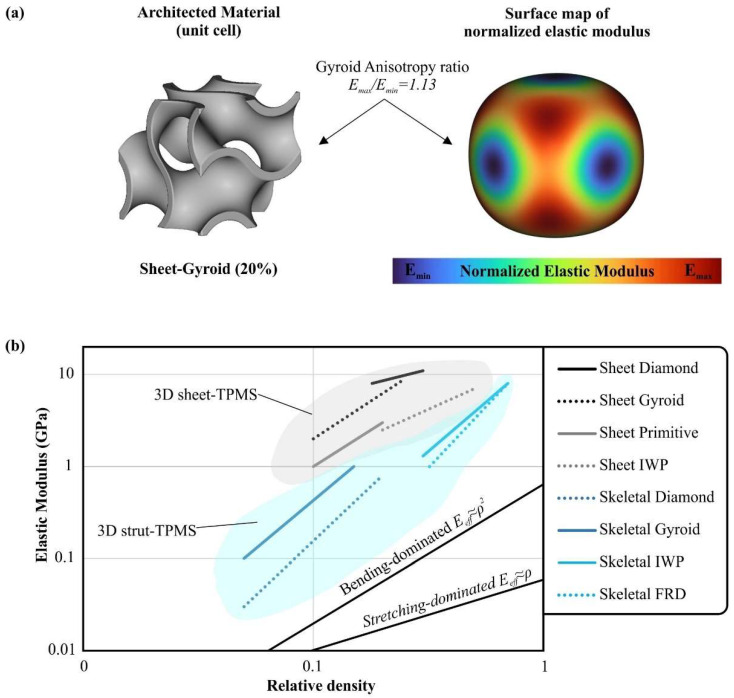
(**a**) Indicative surface map of the normalized local elastic modulus for a Gyroid structure coupled with a value of the *E_max_*/*E_min_* ratio, (**b**) Effective elastic modulus and the mechanical behaviors of several TPMS architected materials constructed with metal additive manufacturing [20,62,63,64,65,66].

For non-cubic architected materials and for construction materials with linear elastic behavior, the anisotropy can be measured with the assistance of the *E*/*E_max_* ratio, where *E* is the local elastic modulus and *E_max_* is the maximum local elastic modulus. In this method, a surface map of local elastic modulus is created for every point of the unit cell in all three directions providing data for the location of the highest anisotropy within the structure. Furthermore, Tancogne-Dejean et. al. [58] has proposed the *E_max_*/*E_min_* ratio in order to measure the anisotropy of the structure. Again, this is a positive dimensionless number with the isotropic structures having a value of one, and as this number increases the higher the anisotropy is. Figure 4a shows an indicative surface map of the normalized local elastic modulus for a Gyroid structure coupled with a value of the *E_max_*/*E_min_* ratio.

After the calculation of the anisotropy of an architected material, the next step is the evaluation of its mechanical behavior. Due to the geometric complexity of the architected materials, it is extremely difficult to calculate its cross-section area, especially for ultra-low relative densities (<5%), as it changes in every layer of the structure. For these reasons, the effective mechanical properties of architected materials are calculated in order to observe their mechanical behavior. The effective mechanical properties are the mechanical properties of the lattice structures, which are calculated utilizing the dimensions (length, area, etc.) of the structure’s bounding box. According to the literature [19], these effective properties comply with the following scaling law:

(11) $$\Phi_{lattice}=\Phi_{solid}\cdot C\cdot {\left(\bar{\rho }\right)}^{n}$$

with *Φ_lattice_* and *Φ_solid_* representing the mechanical property for the lattice structure and solid material, respectively. Regarding the previous equation, *C* and *n* are constants that are dependent on the selected architected material. Furthermore, *ρ_relative_* represents the relative density of the applied structure. The main mechanical properties of an architected material are the effective properties of elastic modulus, yield stress, and peak stress coupled with the structure’s Poisson ratio and energy absorption. In order to extract these properties, quasi-static uniaxial tests are usually employed, as it is shown in the literature that is listed in Figure 4b. Furthermore, the calculation of the scaling law’s constants is performed by testing at least three different relative densities of the same architected material and then the curve-fitting process is utilized. For example, in order to extract the mechanical behavior of an architected material, at least three samples of different relative densities are examined under quasi-static uniaxial loading and the effective mechanical properties are derived from the engineering stress–strain diagrams. Then, for each effective mechanical property, a curve-fitting procedure is performed based on the values for each relative density. It is worth mentioning that the energy absorption of an architected material is calculated by the surface area below the stress-strain diagrams for up to 50% strain before the densification effect occurs [59,60]. Equation (12) describes the mathematical formula of energy absorption.

(12) $$W=\int_{0}^{\varepsilon_{0}}\sigma \left(\varepsilon \right)d\varepsilon$$

The scaling laws are useful tools for architected materials in order to understand their overall mechanical behavior (stretching or bending dominated behavior) and to predict their effective mechanical properties based on the applied relative densities. It is worth noting that according to the literature [19], the scaling laws are applicable to architected materials with relative densities below 50%. As was mentioned in the previous sections, the most essential scaling law of an architected material is the one that concerns the effective elastic modulus (*E_lattice_ = E_solid_ C*(*ρ*)*^n^*). The values of constant *C* are ranging from 0.1 to 4 [28,61]. On the other hand, the values of n are positive numbers that reveal the mechanical behavior of the structure.

Several experimental and numerical published studies examined the mechanical behavior of architected materials, both for strut and sheet structures, and extracted the scaling law constants for examined architected materials made of specific construction material. Table 1 summarizes the results of some of these studies. The majority of these research articles show that architected materials with stretching-dominated behavior (*n* ≈ 1) revealed higher stiffness and superior mechanical strength. In addition, it was observed that structures with a lower *n* value for effective elastic modulus have enhanced mechanical performance compared with structures with stretching-dominated behavior but with a higher *n* value. For example, Neovius and Schwarz Diamond sheet-TPMS architected materials have higher mechanical strength than Octet structure besides the fact that all experienced stretching-dominated behavior. Figure 4b presents the effective elastic modulus and the mechanical behavior of several metal 3D printed TPMS lattices compared with the curves of each mechanical behavior [20,62,63,64,65,66].

Besides the experimental data from the architected material’s mechanical tests, extensive research has been performed in order to build accurate finite element models (FEM), which could simulate or even predict the mechanical response of each architected material. The majority of these studies concern periodic architected materials due to the symmetry of their structure. The symmetric structure facilitates the solution of the solver’s mathematical problem utilizing representative volume elements (RVEs), i.e., the unit cell of the structure, and periodic boundary conditions (PBC). Furthermore, due to the extensive degradation of mechanical properties, elastic, plastic, viscoelastic, and hyperelastic material models have been utilized across the literature [27,28,61,66,67,68,69,70]. The employed material model depends on the construction material and applied relative density, as these two parameters influence both the deterioration of the effective mechanical properties and the elastic/plastic behavior of the structure. In Table 2, a series of published research is listed that utilized different material models to simulate the mechanical response of various architected materials.

## 5. Applications and Future Research of Architected Materials

### 5.1. Current Application of Architected Materials

Architected materials are employed in order to structurally optimize a part’s geometry by reducing the mass and providing a more uniform mass distribution. In the last decade, architected materials have proceeded from objects of scientific research to commercial applications, due to the development of advanced design software and industrial employment of additive manufacturing technologies. More specifically, their unique physical and mechanical properties are utilized in the following series of applications.

The first implementation of architected materials and lattice structures was for parts with lightweight purposes in the automotive and aeronautics industries, due to their high porosity [3,4,5,6]. In detail, architected materials were used first as cores in sandwich-like structures, in order to reduce the mass and maintain strength and stiffness [72,73]. Currently, with the assistance of additive manufacturing technologies, the employment of architected materials is performed with the topology optimization of existing structural parts. For these applications, architected materials with stretching-dominated behavior are usually utilized due to their increased stiffness and peak strength. Thus, the solid regions of the existing parts are removed and replaced with lattice structures with a specific geometry and relative density, in order to achieve the desired mechanical properties.

Furthermore, architecture materials reveal enhanced energy absorption performance, as it was mentioned in the previous sections. Their ability to absorb high amounts of mechanical energy opens the way for many applications with primary objectives the high crashworthiness and the storage and diffusion of energy. For example, lattice structures are utilized for packaging purposes, as they can absorb energy and loads to maintain the product unharmed [19]. Applying the same principle, lattice structures have been embedded within the protection equipment of athletes, such as helmets, splints, etc. Furthermore, due to their high crashworthiness, the architected materials have been proposed as a protection shield in aerospace vehicles [42,60]. Finally, architected materials made of elastomeric materials could be used as absorbers [59]. It is worth mentioning that based on the application and the applied construction material different additive manufacturing techniques could be utilized from Fused Filament Fabrication (FFF), Stereolithography, Material Jetting, Binder Jetting, Selective Laser Sintering (SLS) for polymers and elastomers to Selective Laser Melting (SLM) and Directed Energy Deposition (DED) for metals and alloys.

Besides their mechanical behavior, the architected materials possess two main physical characteristics, which are competitive advantages for a plethora of applications. These characteristics are the high porosity and high surface area to volume ratio, mainly in sheet-architected materials. The high porosity means two things: first, the overall volume is partially filled with materials, and second, the rest of the volume is filled with air or could be filled with different materials. Hence, the existence of air between the pores of the structure increases the thermal insulation of the structure, achieving very low thermal conductivity. For this reason, architected materials (honeycombs) were used in the aerospace industry to thermally insulate the booster rockets of the space shuttle. Moreover, the existence of air in the closed architected materials provides the extra property of buoyancy, which combined with the high-energy absorption and sufficient mechanical strength, become suitable materials for lightweight boats, canoes, etc. In addition, following the same rule, the architected materials with high porosity can be used in acoustic insulation applications, as the cellular structure coupled with air pores leads to a high damping capacity of the structure working as a sound absorber [8,74]. On the other hand, due to the fact that a sufficient amount of volume is practically empty (or filled with air), the architected materials are investigated as 3D printed biomaterials utilizing 3D bio-printer or additively manufactured bio-polymers via a series of additive manufacturing methods (FFF, SLS, etc.). Existing studies [7,8,75,76] have shown that the existence of pores within a structure could accelerate the tissue regeneration process due to the fact that large pores and channels within the structure’s domain facilitate the diffusion of blood and nutrients. Moreover, the high porosity and the high surface area to volume ratio provide the necessary circumstances to position growth factors into the architected materials volume to assist the generation of cells. Furthermore, the high surface area to volume ratio of architected materials, especially sheet lattices, is a unique characteristic that can accelerate chemical and physical processes. Many chemical and physical processes are dependent on the contact surface area or the contact length, such as heat transition and catalysis. In detail, the larger the surface area or the length of the surface increases the speed of the physical or chemical process. Thus, architected materials with a large surface area to volume ratio, such as sheet-TPMS, are utilized for heat exchanger and temperature control applications fabricated with the SLM additive manufacturing method. Furthermore, due to the fact that the same principle governs the chemical reaction, architected materials are also utilized for catalysis and battery application [77].

Other applications of architected materials could be filters and static mixers. These applications are derived from the combinations of the following characteristics: high porosity, high surface area to volume ratio, and high geometrical complexity. The ceramic architected materials have been used as filters in metal casting due to the ability to trap the inclusions with the structure [19]. Static mixers, i.e., mixers without any moving parts, are consisted mainly of sheet-architected materials and mixes two or more fluids through their geometry [78]. Moreover, architected materials deploy high friction forces due to their unique shape and multiple surfaces. Thus, they were employed as carriers of fluids with high viscosity, which control or stop the flow of the fluid without the existence of external force, or as components with high friction in order to achieve tough assembly of components. One of the most successful advantages of this is the high-performance Trilock hip implant via the SLS additive manufacturing technique [79].

### 5.2. Future Research in Architected Materials

The characterization of the mechanical behavior of architected materials and their novel structures is an ongoing research field in order to improve their utilization in the aforementioned commercial applications. However, the most promising and cutting-edge research field of architected materials focuses on 4D printing applications and novel micro-lattices. In detail, 4D printing employs advanced composite materials with unique characteristics [44,80], coupled with architected structures, mainly auxetic structures, in order to achieve shape-shifting structures after the employment of a specific stimulus. In detail, smart materials are usually shape-memory materials that with the proper stimulus, such as electricity, temperature, pH, etc., deploy internal stresses [81]. So, when a smart material is combined with auxetic architected materials, the internal stresses are transformed to structure deformation. Four-dimensional printing is a cutting-edge technology with application in various scientific fields, such as biomedical with a 4D-printed stent, robotics with soft 4D-printed grippers and actuators, advanced electronics, etc. [44,82,83]. On the other hand, micro-fabrication of lattice structures gains more and more scientific interest with multiple potential applications in bioengineering, sensor industry, and microelectromechanical systems (MEMS) [44,84,85]. For these applications, architected materials are fabricated with smart materials and novel additive manufacturing techniques, such as Two-Photon Polymerization (2PP), in order to produce nanostructures customized for the desired application [86].

## 6. Conclusions

In the current review, an extensive analysis of architected materials was presented, as the 3D printing technologies open the way for their commercial applications with topology-controlled and optimized 3D printed parts. More specifically, the theoretical background around the architected materials was described, and the main two classification types were analyzed based on their geometry and mechanical behavior. Furthermore, the most widespread optimization methods for the existing architected materials were listed along with their potential applications. Moreover, the main mechanical properties of the architected materials were presented with the process of evaluating them. In addition, published experimental and numerical results were discussed in order to complete the analysis of the architected materials’ mechanical response. Finally, in the last section, a plethora of the existing applications of 3D printed architected materials and their potential applications were presented and analyzed. To conclude, architected materials and additive manufacturing are the future of topology-controlled and optimized components; thus, this review establishes a detailed guide of the basic characteristics of the architected materials. However, there is a lot of ground that should be covered in extended mechanical properties of architected materials, such as fatigue, creep, etc., before their widespread commercial and industrial use.

## Figures and Tables

**Table 1:** Scaling laws constants for different architected materials from published studies.

Study	Construction Material	Structure	Elastic Modulus		Strength	
			*C*	*n*	*C*	*n*
[62]	Photopolymer resin	Schwarz Primitive	0.871	1.437	0.923	1.769
		Gyroid	0.952	2.174	0.618	1.746
		Schwarz Diamond	0.644	2.026	0.68	1.614
		IWP	1.204	2.654	0.879	2.124
[27,28]	Polyamide 12 (PA12)	Kelvin	3.06	3.5	12.75	4.22
		Rhombic Dodecahedron	0.76	2.63	2.12	2.83
		Waeire-Phelan	0.3	1.7	0.76	1.79
		Octet	0.13	1.11	0.62	1.6
		Schwarz Primitive	0.4	2.01	1.61	2.55
		Gyroid	0.16	1.22	0.34	1.14
		Schwarz Diamond	0.43	1.2	0.37	0.91
		Neovius	0.42	1.2	0.34	0.77
[20]	Maraging steel	Kelvin	0.15	1.56	1.375	1.83
		Octet	0.1	1.23	0.615	1.32
		Gibson-Ashby	0.15	1.62	1.185	1.76
		Skeletal-IWP	0.19	2.01	0.153	2.17
		Skeletal-Diamond	0.35	2.22	4.418	2.73
		Skeletal-Gyroid	0.14	1.68	1.189	1.86
		Sheet-IWP	0.08	1.15	2.354	2.13
		Sheet-Diamond	0.037	0.522	0.933	1.39
		Sheet-Gyroid	0.1	1.23	0.885	1.43
		Sheet-Primitive	0.11	1.31	1.419	2.13

**Table 2:** Numerical studies with different applied material models.

Study	Material Model	Construction Material
[61,68]	Elastic	PA2200, Polymer composites
[67,69]	Elastic-Plastic	Titanium alloys, Photopolymer resins
[66,70]	Elastic-Viscoelastic	Ti6Al4V, PA2200
[27,28,71]	Hyperelastic	PA12, PA2200

## Data Availability

Not applicable.
